# The value of virological research in wildlife

**DOI:** 10.1128/mbio.03616-25

**Published:** 2026-02-18

**Authors:** Anice C. Lowen, Jonathan A. Runstadler, Seema S. Lakdawala

**Affiliations:** 1 Department of Microbiology and Immunology, Emory University School of Medicine 12239, Atlanta, Georgia, USA; 2 Department of Infectious Disease and Global Health, Cummings School of Veterinary Medicine, Tufts University 146186, Boston, Massachusetts, USA; Tsinghua University, Beijing, China

**Keywords:** virology, surveillance, wildlife, pandemic preparedness

## LETTER

In a recent article published in *mBio* (1), Aatresh and Lipsitch consider the utility of research aimed at the discovery of novel viruses in wildlife. They term this activity ‘viral prospecting’ and investigate its benefits by testing two narrow hypotheses: (i) that viral prospecting in animals identifies potential viral causes of outbreaks in humans; and (ii) that it translates to accelerated vaccine development. Based on their findings, Aatresh and Lipsitch question the value of virus discovery.

We have three primary concerns with their work. First, the premise that the contributions of viral discovery to preparedness can be meaningfully assessed by considering vaccine development is unsound. Second, the arguments made overlook the profound benefits of fundamental research. Third, the article does not distinguish carefully enough between efforts specifically aimed at discovering new viruses and field work designed to elucidate the changing ecology and evolution of known viruses, leaving room for misinterpretation. We discuss each of these concerns in more depth below.

Focusing on vaccine development as an endpoint sets an extremely high bar for evaluating the utility of field research for pandemic preparedness. In fact, the analytic strategy of focusing on this singular outcome assures the conclusion that viral discovery has minimal impact. Medical countermeasures can take many years to develop, but much of the analysis Aatresh and Lipsitch describe was limited to the last 20 years. As the authors highlight, vaccine development remains an elusive goal, even for many high-priority viruses. This reality reflects the technical and regulatory challenges of advancing a candidate vaccine to human use (2). Since evaluation of efficacy is not possible, the barriers to clinical testing are especially acute for a pathogen that is not in circulation (3). In effect, using licensed vaccines as a metric to assess the value of virus discovery is like judging the benefits of a physical training regimen by the number of Olympic medals it has produced. Viral discovery is not designed to produce vaccines, just as most training programs are not intended to produce Olympic performance. But, each has importance for a broader goal of improving public health readiness and individual health.

While the expectation that licensed vaccines will follow from virus discovery is unrealistic, prior knowledge and isolation of a virus can enable timelier public health responses than would be possible if its discovery coincides with its spillover into humans. In the best case, field studies may allow risk mitigation to be implemented in non-human populations, with much smaller societal impact compared to measures designed to prevent transmission following spillover into humans (3–5). With knowledge gained through virus discovery, outbreak preparation can also be furthered through predictive modeling, development of actionable response policies, and validation of diagnostic and therapeutic modalities (6–8). Additionally, detection of a potential pathogen in wildlife can permit steps that precede the production of a licensed vaccine, such as selection of representative strains or design of stabilized immunogens (9, 10). Thus, while outcomes may be less tangible than a licensed vaccine, what we learn from virological research in wildlife is fundamental to outbreak preparedness. Furthermore, successful discovery and follow-on research may actually reduce the impetus for vaccine development.

Beyond the sharp focus on vaccines as a measure of preparedness, an important concern with the article contributed by Aatresh and Lipsitch lies in its potential to be understood as an argument against all virological research in wildlife. Given limited resources, we agree that research that can inform public health should be prioritized. There are indeed many potential zoonotic viruses in wildlife, and, at present, simple cataloging of these likely has little direct public health benefit. However, field research that is targeted to high-risk situations is an extremely important feature of outbreak preparedness and response: we need to know when new highly pathogenic influenza virus strains are sweeping through migratory birds so that we can protect farm animals and farm workers by increasing awareness and biosecurity (11). We need to identify bat colonies that harbor Marburg virus to inform risk communication in local communities (12–14). We need to understand patterns of hantavirus prevalence in rodents to calibrate efforts that prevent human exposure (15). We need to understand how ongoing viral evolution is producing new viruses from known foes (16). While Aatresh and Lipsitch exclude surveillance for known zoonotic pathogens from their analyses, we worry that many readers will understand from the article that work in wildlife should be discouraged in service of research in humans or at human–animal interfaces. This is deeply concerning because, as we have argued previously (17), it is essential for humanity’s efforts to combat viral threats that the linkages among wildlife, the environment, and humans are recognized as fundamental to pandemic emergence, by the virology community, by funders, and by the public.

Finally, through their evaluation of virus discovery, Aatresh and Lipsitch disregard the value to society of research undertaken at least in part with the goal of discovery, rather than application. Fundamental research enriches humanity, fuels science, and forms the foundation for innovations that improve human health and well-being. These benefits are well-established (18, 19), and they extend to field research resulting in viral discovery. While the tangible benefits of fundamental research often cannot be anticipated, one example is the value of understanding the extent to which traits or functions are conserved across diverse viruses, and, therefore, which could serve as targets for broadly applicable interventions (20, 21). Another is the value of understanding the context of exposure in assessing the risk of zoonotic viruses (22, 23).

Research is our best defense against pandemic threats. Within this, a deep understanding of viral ecology and evolution in reservoir hosts is critical: this research can define the processes that drive emergence and circulation of novel viruses and identify host species and ecological conditions that increase the risk of human outbreaks. Virus discovery in wildlife may be unlikely to accelerate vaccine development in the short term, but it can inform and initiate local action that reduces risk to humans in the immediate term. Positive impacts of surveillance research are manyfold, and increasingly important as changing ecology and human activity expand zoonotic disease.
